# The Region Centromeric to *HLA-C* Is a Key Region for Understanding the Phenotypic Variability of Psoriatic Arthritis

**DOI:** 10.1155/2014/570178

**Published:** 2014-01-30

**Authors:** Rubén Queiro, Patricia Tejón, Sara Alonso, Pablo Coto, Carlos López-Larrea, Jesús Martínez-Borra, Segundo González

**Affiliations:** ^1^Rheumatology Department, Hospital Universitario Central de Asturias (HUCA), C/Celestino Villamil S/N, 33006 Oviedo, Spain; ^2^Dermatology Department, HUCA, C/Celestino Villamil S/N, 33006 Oviedo, Spain; ^3^Immunology Department, Histocompatibility Unit, HUCA, C/Celestino Villamil S/N, 33006 Oviedo, Spain; ^4^Department of Functional Biology, IUOPA, University of Oviedo, C/Celestino Villamil S/N, 33006 Oviedo, Spain

## Abstract

With the aim of clarifying the role of several polymorphisms around the *HLA-C* locus in the clinical expression of PsA, the distribution of several polymorphic markers and genes located around the *HLA-C* locus was analyzed in a well-established cohort of 110 patients with PsA, 50 patients with psoriasis alone, and 110 healthy controls. The frequency of these genes was also analyzed by PsA articular models, based on three main subgroups: oligoarthritis, polyarthritis, and spondylitis. Distal interphalangeal joint (DIP) involvement was associated with the presence of *MICB-CA20* (OR 6.0, 95% CI: 1.58–22.69, *P* = 0.005). *HLA-DRB*∗*07* was associated with oligoarticular forms of PsA (OR 4.1, 95% CI: 1.8–9.3, *P* = 0.0007). The spondylitic forms overexpressed the antigen *HLA-B*∗*27* (OR 5.7, 95% CI: 2.4–13.6, *P* = 0.0001). *MICA-A5.1* showed association with polyarthritis (OR 3.7, 95% CI: 1.5–8.8, *P* = 0.006). Genes telomeric to *HLA-C* were overexpressed in psoriasis but not in PsA subphenotypes. This study shows that the region centromeric to *HLA-C* is a key region that expresses not only disease risk genes but also genes that help explain the phenotypic variability of PsA.

## 1. Introduction

Psoriatic arthritis (PsA) is a complex disease in which environmental, host, and random factors interact resulting in disease in genetically susceptible individuals [1]. This condition is associated with significant morbidity and mortality and is estimated to result in a cost to healthcare systems equivalent to that of rheumatoid arthritis [2]. Hence, the identification of the etiological factors would be an important advance because it may suggest therapeutic targets for the development of specific drugs and help establish the prognosis of patients.

**Figure 1:**
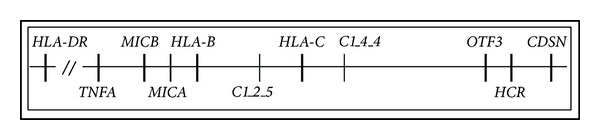
Map of the *MHC* region. Polymorphisms in microsatellite *C1_4_4*, octamer transcription factor 3 gene (*OTF3*), *a*-helix coiled-coil rod homologue (*HCR*), corneodesmosin gene (*CDSN*), *HLA-C*, microsatellite *C1_2_5, HLA-B, MICA, MICB, TNFA*, and *HLA-DRB1* were analyzed in this study.

Linkage and association analyses have shown that the major histocompatibility complex (*MHC*) is the major genetic determinant related to psoriasis susceptibility. Within the *MHC*, HLA-C*06 is the allele that shows the strongest association with psoriasis [3, 4]. Other *HLA* class I alleles associated with the disease (i.e., *B13, B47,* and *B57*) are due to linkage disequilibrium with HLA-C*06 [3, 4]. The association of *HLA* with PsA is more complex than that found in psoriasis. Although HLA-C*06, B*13, and B*57 have also been associated with PsA, the association is much weaker and seems to be related to psoriasis rather than arthritis [3, 4]. Centromeric to *HLA-C*, a susceptibility gene (*MICA*) has been associated with arthritis risk independently of C*06, though other authors have not confirmed this finding [5, 6]. Other nearby genes that are in linkage disequilibrium with *HLA* class I or *MICA* could determine susceptibility to PsA or further increase the risk of developing the disease. For instance, HLA-B*38 and B*39 are associated with peripheral polyarthritis whereas HLA-B*27 is associated with spinal involvement [3, 4, 7]. *HLA* class II antigens have also been associated with psoriasis and PsA. Thus, associations have been described between HLA-DRB*07 and psoriasis and between DRB*04 and PsA, although these associations have not been confirmed in all populations studied [3, 4, 7]. Furthermore, *HLA-DR17* (corresponding to HLA-DRB*03) has been recently linked to psoriatic enthesopathy [8]. However, it is not clear whether these alleles may be associated with a specific phenotype of some PsA patients rather than the development of the disease since genetic studies in PsA are complicated by the clinical heterogeneity of the disease. Consequently, stratification of patients by clinical subset may be useful in reducing the heterogeneity of the disease in *MHC* association studies and may help to identify true genetic and prognostic markers of the disease. In the present report we analyzed polymorphisms of several genes centromeric and telomeric to *HLA-C* (Figure 1) to assess the potential relationship between them and the presence of different articular subphenotypes in a well-established cohort of PsA patients.

## 2. Patients and Methods

### 2.1. Patients

One hundred and ten consecutive patients who met the classification criteria for psoriatic arthritis (CASPAR) [9] were consecutively selected from those attending the rheumatology outpatient clinic of a tertiary care institution. There were 55 men and 55 women with a mean age of 49 ± 12 years. The mean disease duration for psoriasis was 19 ± 10 years and 13 ± 8 years for arthritis. Psoriasis preceded arthritis in 75% of cases and a family history of psoriasis was recorded in 42% of patients. Patients were initially classified according to the Moll and Wright model [10] and thereafter according to the predominant articular pattern seen in the last 5 years of followup. In accordance with the main clinical and radiographic features of the last 5 years of disease evolution, 42 patients showed an oligoarticular pattern (swollen joint count—SJC—≤4), 30 had polyarthritis (SJC ≥ 5), and 38 had predominant axial disease (radiographic sacroiliitis plus inflammatory back pain irrespective of the presence of peripheral synovitis). The involvement of distal interphalangeal joints (DIP) as well as the “mutilans forms” was recorded as typical features of PsA but not as independent models of the disease.

This study was performed in accordance with the Declaration of Helsinki. All patients and controls gave their written informed consent before participating in the study. The study was conducted after approval by the ethics committee of our hospital.

### 2.2. *HLA* Typing

*HLA-C* was typed using the polymerase chain reaction with sequence-specific primers (PCR-SSP). Polymorphisms of the octamer transcription factor 3 gene (*OTF3*), the corneodesmosin gene (*CDSN*), and the *a*-helix coiled-coil rod homologue (*HCR*) gene were analyzed as previously described [11]. Microsatellites *C1_2_5* and *C1_4_4* were amplified using the PCR primers reported by Tamiya et al. [12]. *HLA-B* typing was performed using the Dynal RELI SSO *HLA-B* test following manufacturer's instructions. *HLA-DRB1* alleles were typed and subtyped using a polymerase chain reaction with specific primers (PCR-SSP). For the analysis of microsatellite repeat polymorphism in the *MICA* gene, PCR was performed out using primers labeled at the 5′ end with the fluorescent reagent Cy5 as previously described [11]. For *MICB* typing, a dinucleotide microsatellite polymorphism in the first intron was analyzed by PCR, and *TNF-*α** polymorphisms at positions −238 and −308 were typed by PCR as previously described [11].

All previous typing was also performed in 50 patients suffering psoriasis alone as well as in a control population of 110 randomly selected ethnically and geographically matched blood donors.

### 2.3. Statistical Analysis

Differences between the frequencies of these allelic markers in patients and controls and differences found depending on the articular patterns previously described were assessed using a Chi-square test with Yates's correction and Student's *t*-test. The odds ratio (OR) was calculated by the cross-product ratio and 95% confidence intervals by the Cornfield method. The extent of linkage disequilibrium between the two loci is expressed as the observed disequilibrium value (*λs*), that is, a proportion of the theoretical maximum disequilibrium value (*λ*_max⁡_) achievable for this combination of alleles. The *λs* values were calculated using the following formula: *λs* = *λ*/*λ*_max⁡_ = Pab − (Pa · Pb)/Pa · (1 − Pb).

## 3. Results

HLA-C*06 was increased in both populations, whereas *MICA-A9 *was increased only in arthritic patients (Table 1). Patients suffering psoriasis alone showed a higher frequency of HLA-C*06, C1_4_4 microsatellite, *OTFHind3 *gen, and *HCR* gen. However, genes centromeric to *HLA-C* were not overexpressed in psoriatic patients (Table 1).

The analysis of the distribution of these markers according to the articular patterns defined in the study disclosed significant associations between the forms with DIP involvement and *MICB-CA20*. Of a total of 50 patients with DIP involvement, 12 had this allele compared to only 3 of 60 without this type of articular involvement (24% versus 5%, OR 6.0, 95% CI: 1.58–22.69, *P* = 0.005). HLA-DRB*07 was overexpressed in patients with oligoarticular forms (59.5%) as compared to polyarticular (13.3%) and axial forms (36.8%), OR 6.1, 95% CI: 2–17, *P* = 0.0007. The axial forms overexpressed the antigen HLA-B*27 (57.9% versus 23.8% in oligoarthritis and 13.3% in polyarthritis), OR 5.7, 95% CI: 2.4–13.6, *P* = 0.0001. *MICA-A5.1* showed association with polyarthritis (56.7%) but not with oligoarthritis (21.4%) nor with axial forms of PsA (31.6%), OR 3.7, 95% CI: 1.5–8.8, *P* = 0.006 (Table 2). Genes located telomeric to *HLA-C* did not contribute to the differentiation PsA into articular subtypes.

## 4. Discussion

Characterization of the exact *MHC* gene or genes involved in susceptibility to psoriasis and psoriatic arthritis has been controversial. This is due to the high density of polymorphic genes located in this region, the extensive ranges of polymorphism, and the preservation of *HLA* haplotypes [13]. For the same reasons, to date it has been difficult to link the various joint patterns of PsA to specific genes in this region. Many of the associations between *MHC* genes and joint forms of PsA invoked in the past are better explained by the known phenomenon of linkage disequilibrium between these and HLA-C*06 or by a specific association with the phenotypic characteristics of the disease [3, 4, 7].

In this study, the analysis of a wide *MHC* sector disclosed that several genetic variants of this region contribute to both the risk of disease and the differentiation of the various articular subphenotypes of the disease. As a whole, our results point to the region telomeric to *HLA-C* as being important for psoriasis development, whereas the centromeric region seems to play a key role in susceptibility and disease expression in PsA.

In the present report, the region centromeric to *HLA-C* not only has been confirmed as a key arthritogenic region (*MICA-A9*) but also appears to contribute to the varied joint phenotype expression of PsA (HLA-DRB1*07, HLA-B*27, *MICB-CA20*, and *MICA-A5.1*). The association of *MICA-A5.1* with polyarthritis and of *MICB-CA20* with DIP involvement has not been highlighted before. Therefore, *MICA *and *MICB *regions appear as two of the most relevant regions, among those centromeric to *HLA-C*, to explain PsA articular phenotype variability. Though the role of *MICA* and *MICB* polymorphisms in the pathogenesis of PsA has yet to be elucidated, the potential involvement of these genes in the risk and expression of the disease makes the role of innate immunity significant in the pathogenesis thereof [14, 15]. *MICA* is a polymorphic gene capable of activating NK and T cells via NKG2D. Deregulation of *MICA* expression has been implicated in the pathogenesis of rheumatoid arthritis and other T cell-mediated autoimmune diseases; therefore it should not be surprising that these genes have a role in the pathogenesis of PsA [14, 15]. Some of our findings support those of the previous literature, such as the relationship between *HLA-B27* and spondylitic variants of PsA or of *HLA-DR7* with less serious forms of the disease [16, 17]. The role of *MICA* genes in PsA susceptibility remains, however, controversial since some investigations have corroborated it, but others not [6, 18]. The potential relationship between *HLA-B27* and DIP involvement invoked in the past has not been consistent (we could not show it here); however, the potential link between DIP involvement and a gene such as *MICB* fits more easily with the current pathogenic model of the entheso-synovial organ of the disease, where factors related to innate immunity outweigh those of adaptive immunity [19].

## 5. Conclusion

This study confirms previous association of HLA-C*06 and *MICA-9* with PsA susceptibility; however it also suggests a complex interaction between genes located centromeric to *HLA-C* and the phenotypic characteristics of the disease that could be relevant to the prognosis and treatment of this condition.

## Figures and Tables

**Table 1:** Major polymorphic variants found in this study in patients with psoriasis (PsO) and psoriatic arthritis (PsA).

Marker	PsO *n* = 50	Controls *n* = 110	*P* values	PsA *n* = 110	Controls *n* = 110	*P* values
*HL* *A*-C*06	25 (50%)	19 (17%)	<0.00001^a^	62 (56.4%)	19 (17%)	<0.00001^e^
*MICA-A9 *	15 (30%)	33 (30%)	NS	66 (60%)	33 (30%)	<0.00001^f^
*MICA-A5.1 *	18 (36%)	44 (40%)	NS	38 (34.5%)	44 (40%)	NS
*MICB-CA20 *	5 (10%)	9 (8.1%)	NS	15 (13.6%)	9 (8.1%)	NS
*HL* *A*-*DRB*1*07	15 (30%)	33 (30%)	NS	43 (39.1%)	33 (30%)	NS
*HL* *A*-B*27	5 (10%)	8 (7.3%)	NS	36 (32.7%)	8 (7.3%)	0.001^g^
*C1_4_4 (384) *	28 (56%)	25 (23%)	0.0001^b^	60 (54.5%)	25 (23%)	0.0001^h^
*OTF3 Hind3 *	42 (84%)	66 (60%)	0.0033^c^	73 (66.4%)	66 (60%)	NS
*HCR (Pg8) *	31 (62%)	28 (25.5%)	<0.00001^d^	37 (33.6%)	28 (25.5%)	NS

^a^OR 4.8 (2.3–10.1).

^b^OR 4.3 (2.1–8.8).

^c^OR 3.5 (1.5–8.01).

^d^OR 4.8 (2.3–9.7).

^e^OR 6.18 (3.32–11.51).

^f^OR 3.5 (2.0–6.12).

^g^OR 5.9 (2.6–13.4).

^h^OR 4.3 (2.2–8.4).

There was linkage disequilibrium between *HLA*-C*06 and *C1_4_4 * (*λs* = 0.6).

**Table 2:** Distribution of the main genetic markers according to the articular patterns defined in the study. Psoriatic arthritis population: 110.

Marker	Oligoarthritis *n* = 42 (%)	Polyarthritis *n* = 30 (%)	Axial disease *n* = 38 (%)	*P* values
*HL* *A*-C*06	22 (52.4)	12 (40)	18 (47.4)	NS
*MICA-A9 *	27 (64.3)	17 (56.7)	22 (57.9)	NS
*HL* *A*-*DRB*1*07	25 (59.5)	4 (13.3)	14 (36.8)	*P* < 0.0007*
*HL* *A*-*DRB*1*04	5 (12)	4 (13.3)	5 (13.1)	NS
*HLA-B27 *	10 (23.8)	4 (13.3)	22 (57.9)	*P* < 0.0001**
*TNF-238A *	6 (14.3)	5 (16.7)	8 (21)	NS
*TNF-238G *	34 (81)	25 (83.3)	29 (76.3)	NS
*TNF-308A *	12 (28.6)	9 (30)	12 (31.6)	NS
*TNF-308G *	28 (66.7)	21 (70)	25 (65.8)	NS
*MICA-A5.1 *	9 (21.4)	17 (56.7)	12 (31.6)	*P* < 0.006***

*OR 4.1 (1.8–9.3). **OR 5.7 (2.4–13.6). ***OR 3.7 (1.5–8.8). NS: nonsignificant. Note: significant *P* values represent intergroup comparisons.
